# P53 independent pathogenic mechanisms contribute to BubR1 microcephaly

**DOI:** 10.3389/fcell.2023.1282182

**Published:** 2023-10-12

**Authors:** Noelle A. Sterling, Bethany K. Terry, Julia M. McDonnell, Seonhee Kim

**Affiliations:** ^1^ Shriners Hospitals Pediatrics Research Center, Department of Neural Sciences, Lewis Katz School of Medicine, Temple University, Philadelphia, PA, United States; ^2^ Biomedical Sciences Graduate Program, Lewis Katz School of Medicine, Temple University, Philadelphia, PA, United States

**Keywords:** BubR1, mitosis analysis, neural progenitor, p53, microcephaly, cortical development, DNA damage

## Abstract

The mosaic variegated aneuploidy (MVA)-associated gene *Budding Uninhibited by Benzimidazole 1B* (*BUB1B*) encodes BUBR1, a core member of the spindle assembly checkpoint complex that ensures kinetochore-spindle attachment for faithful chromosome segregation. *BUB1B* mutation in humans and its deletion in mice cause microcephaly. In the absence of BubR1 in mice, massive cell death reduces cortical cells during neurogenesis. However, the molecular and cellular mechanisms triggering cell death are unknown. In this study, we performed three-dimensional imaging analysis of mitotic BubR1-deficient neural progenitors in a murine model to show profound chromosomal segregation defects and structural abnormalities. Chromosomal defects and accompanying DNA damage result in P53 activation and apoptotic cell death in *BubR1* mutants. To test whether the P53 cell death pathway is responsible for cortical cell loss, we co-deleted *Trp53* in BubR1-deficient cortices. Remarkably, we discovered that residual apoptotic cell death remains in double mutants lacking P53, suggesting P53-independent apoptosis. Furthermore, the minimal rescue of cortical size and cortical neuron numbers in double mutant mice suggests the compelling extent of alternative death mechanisms in the absence of P53. This study demonstrates a potential pathogenic mechanism for microcephaly in MVA patients and uncovers the existence of powerful means of eliminating unfit cells even when the P53 death pathway is disabled.

## 1 Introduction

Mosaic variegated aneuploidy (MVA) is a rare autosomal recessive disorder characterized by growth retardation, predisposition to cancer, and microcephaly (Garcia-Castillo et al., 2008; Suijkerbuijk et al., 2010; Bianchi et al., 2018; Pavone et al., 2022). Premature chromatid separation resulting in cells with numerically abnormal chromosomes (aneuploidy) is a hallmark feature of MVA. A well-characterized MVA causative gene, *Budding Uninhibited by Benzimidazole 1B* (*BUB1B*) encodes the BUB-related 1 (BubR1) protein (Bolanos-Garci and Blundell, 2011). Previous work in mice found that a *BubR1* hypomorphic allele substantially reduces protein level presenting with premature aging, and tumor formation, but not microcephaly (Baker et al., 2004; Hanks et al., 2004; Matsuura et al., 2006; Frio et al., 2010; Suijkerbuijk et al., 2010; Miyamoto et al., 2011; Baker et al., 2012; Wijshake et al., 2012; Choi et al., 2016; Yang et al., 2017; Pavone et al., 2022). Successful microcephaly modeling has been shown in *Emx1-Cre*-mediated genetic ablation, which nearly completely depletes BubR1 in cortical progenitors and recapitulates microcephaly phenotypes observed in humans (Simmons et al., 2019).

Microcephaly is a condition diagnosed in children born with head circumferences two or more standard deviations below the mean (Hanzlik and Gigante, 2017). Many genetic causes of microcephaly in humans have been linked to genes required for cell cycle progression, DNA replication and repair, and faithful mitosis (Woods et al., 2005; Faheem et al., 2015). BubR1 is a multidomain spindle assembly checkpoint (SAC) protein that ensures mitotic spindle attachment and prevents premature activation of the anaphase promoting complex (APC) (Mao et al., 2003; Burke and Stukenberg, 2008; Bolanos-Garcia and Blundell, 2011; Suijkerbuijk et al., 2012). Accordingly, *BubR1* loss or mutation can cause microcephaly when impaired checkpoint function results in aneuploidy and subsequent cortical cell loss (Izumi et al., 2009; Malureanu et al., 2009; Lara-Gonzalez et al., 2011; Suijkerbuijk et al., 2012). Previous studies of BubR1 deficiency have described aneuploidy associated with the presence of lagging chromosomes and mitotic slippage (Matsuura et al., 2006; Frio et al., 2010). However, it remains to be determined whether BubR1 microcephaly models indeed have chromosome segregation defects that have been associated with BubR1 depletion and microcephaly pathology.

In *BubR1* conditional knockout (cKO) mice, massive cell death produces significantly smaller cortices consistent with microcephaly pathology (Simmons et al., 2019). In other systems, apoptosis due to genomic alterations or mitotic disruption is often caused by activation of the tumor suppressor protein P53 (Ha et al., 2007; Ohashi et al., 2015; Soto et al., 2017). P53 activation can arrest the cell cycle or initiate apoptosis to eliminate genomically unfit cells as part of its role in mitotic surveillance (Pilaz et al., 2016; Soto et al., 2017; Hafner et al., 2019; Navarro et al., 2020). Importantly, P53 activation has been observed in a variety of microcephaly models (Frank et al., 2000; Gao, et al., 2000; McConnell et al., 2004; Insolera et al., 2014; Marjanovic et al., 2015; Ghouzzi et al., 2016; Mao et al., 2016; Bianchi et al., 2017; Sgourdou et al., 2017; Little and Dwyer, 2019; Shi et al., 2019; Phan et al., 2021; White et al., 2021; Martins et al., 2022; Peek et al., 2022; Sterling et al., 2023).

In some studies, genetic deletion of *Trp53* rescues cortical size, suggesting that P53 activation triggers extensive cortical cell death that leads to microcephaly (Insolera et al., 2014; Marjanovic et al., 2015; Mao et al., 2016; Bianchi et al., 2017; Little and Dwyer, 2019; Shi et al., 2019; Phan et al., 2021; Peek et al., 2022; Sterling et al., 2023). Therefore, it is plausible that mitotic disruption due to BubR1 loss may result in P53 activation and subsequent cell death. Alternatively, in other microcephaly models, P53 elimination does not rescue microcephaly due to the persistence of compounding factors with cellular consequences that are not mediated by P53 (Frank et al., 2000; Gao, et al., 2000; White et al., 2021). To date, *Trp53* co-deletion to determine whether P53 activation mediates BubR1 microcephaly pathogenesis has not been tested.

In this study, we use a *BubR1* cKO mouse model to examine the cellular mechanism underlying the massive cell death that occurs when BubR1 is lost during cortical development. From three-dimensional analysis of mitotic neural progenitors in the developing cortex, we observe that BubR1 loss causes extensive DNA damage and chromosome structural abnormalities. These changes underlie massive cell death, leading to microcephaly. As has been described in other microcephaly models, we observe P53 activation in response to BubR1 cortical loss. However, genetic co-deletion of *BubR1* and *Trp53* only mildly improved microcephaly and cell death without rescuing DNA damage. Importantly, our results implicate a P53-independent cell death mechanism in microcephaly resulting from BubR1 loss. Our study reveals that P53-independent cell death pathways can provide redundancy to ensure the elimination of unfit cells from the cerebral cortex.

## 2 Materials and methods

### 2.1 Animal care and breeding

The experiments described within this manuscript were approved by and performed following the guidelines established by the Institutional Animal Care and Use Committee (IACUC) at the Temple University School of Medicine. Mice were kept on a 12-h light: 12-h dark cycle and provided food and water *ad libitum*. Animals were kept at ambient temperatures between 70 and 74°C and humidity from 30% to 70%. For every experiment, 3–11 animals were analyzed. Both male and female mice were used in these studies.

*BubR1* ^H/H^ mice (Baker et al., 2004) were bred with *Emx1-Cre* mice (Jackson Lab #005628) to generate *BubR1* conditional knockout (cKO) animals (Gorski et al., 2002). *BubR1* cKO animals were crossed with *Trp53* ^*fl/fl*^ animals (The National Cancer Institute Mouse Repository # 01XC2) to generate *BubR1*;*Trp53* double conditional knockout (dcKO) mice. Genotyping was undertaken as previously described using the primers below (Armstrong et al., 1995; Jonkers et al., 2001; Baker et al., 2004).

*Cre*- (F: GCA TTA CCG GTC GAT GCA ACG AGT GAT GAG, R: GAG TGA ACG AAC CTG GTC GAA ATC AGT GCG).

*BubR1-* (F: GTA AGT CTA TTT CTC CTG GAT TAA GTA G, R: CAT CTG TGT ACC ATA CGT GTG TCT GG, R: ATA TTG CTG AAG AGC TTG GCG GCG).

*Trp53*- (F: CAC AAA AAC AGG TTA AAC CCA G, R: AGC ACA TAG GAG GCA GAG AC).

### 2.2 Apical explant staining

At embryonic day (E)14.5, the mouse dorsal cortex was dissected and incubated in 4% paraformaldehyde at 4°C overnight. Samples were then transferred to phosphate-buffered saline (PBS) and maintained at 4°C until staining. Antigen retrieval was performed in 1% sodium dodecyl sulfate (SDS) in PBS for 5 min, followed by rinsing in PBS. Permeabilization was then performed in 0.1% TritonX-100 in PBS for 15 min, followed by PBS washes. Next, samples were incubated in primary antibodies with 5% normal goat serum (NGS) at 4°C overnight and washed again in PBS the next morning. Samples were incubated in secondary antibody solution (Cy3 anti-mouse, Alexa Fluor 647 anti-rabbit) with 5% NGS and Hoechst 33258 at room temperature for 1 h. Samples were washed in PBS and mounted in Fluoromount G (SouthernBiotech #0100-01) on glass slides with the apical face of the cortex oriented upward. Finally, samples were imaged with a confocal microscope (SP8, Leica) in 0.5 μm z-stacks of a 20 μm thickness. Image analysis was performed using LAS AF (Leica) and Photoshop (Adobe v22.2.0). Mitotic cell modeling was performed using Imaris software to create surface objects where samples showed DAPI-labeled DNA.

### 2.3 Immunohistochemistry

Animals were sacrificed at E14.5 and postnatal day (P)21, then perfused with phosphate-buffered saline (PBS) and 4% paraformaldehyde in PBS. Brains were dissected out and fixed overnight in 4% paraformaldehyde in PBS. Collected cortices were embedded in paraffin before microtome sectioning at a thickness of 7 μm.

For immunohistochemistry, cortex sections were rehydrated in a series of 100% xylene and 100%–30% ethanol washes before antigen retrieval in a 10 mM sodium citrate solution. Slides were incubated in primary antibody mixes with the antibodies of interest (information included below) and 5% normal goat serum in PBS at 4°C overnight. After PBS washes the next day, slides were incubated in secondary antibodies (Alexa Fluor 488 anti-mouse, Alexa Fluor 488 anti-rabbit, Alexa Fluor 488 anti-rat, Cy3 anti-rabbit, Cy3 anti-mouse, Alexa Fluor 647 anti-rabbit), 1:500 Hoechst 33342 (Invitrogen #H21492), and 5% normal goat serum in PBS for 3 h. Slides were then washed and mounted with Fluoromount-G (SouthernBiotech #0100-01). TUNEL staining was performed using the Click-iT Plus TUNEL Assay Kit with Alexa Fluor 594 (Invitrogen #C10617).

### 2.4 Primary antibodies

The following antibodies were used for immunohistochemistry: BrdU (ab6326, Abcam, 1:200), Cleaved Caspase-3 (#9661, Cell Signaling Technology, 1:200), CTIP2 (ab18465, Abcam, 1:200), CUX1 (11733-1-AP, Proteintech, 1:200), F4/80 (28463-1-AP, Proteintech, 1:200), FOXP2 (ab16046, Abcam, 1:200), MPM2 (05-368, Cell Signaling, 1:200), P21 (#27296-1-AP, Proteintech, 1:200), PAX6 (#901301, Biolegend, 1:200), PH3 (H0412, Millipore Sigma, 1:500), SOX9 (AB5535, Millipore Sigma, 1:500), TBR2 (ab23345, Abcam, 1:200), and γH2AX (05-636-I, Millipore Sigma, 1:200).

### 2.5 Histology

Hematoxylin and eosin staining was undertaken on the 7 μm paraffin-embedded sections, collected as described in the immunohistochemistry section above. Briefly, samples were rehydrated in a series of xylene and ethanol washes before incubation in hematoxylin and eosin (Scytek #NC0510871). Slides were washed again with another series of xylene and ethanol washes and then mounted with Cytoseal™ 60 (Thermo Scientific #8310-4).

### 2.6 Cell counting and cortical size analyses

For cortical and hippocampal measurements (including length, thickness, and area) at E14.5 and P21, cortical measurements were made using non-consecutive H&E-stained sections for each animal using ImageJ (FIJI) (Schindelin et al., 2012). Cortical surface area measurements were taken from whole brain images using ImageJ (FIJI). Cell counting analyses were completed using Photoshop (Adobe v 22.2.0.). At E14.5, cells were counted within a 200 μm width of cortex, counting all positive cells within the entire height of the cortex. Particle analysis of P21 expression was performed in a 200 μm width of the cortex using ImageJ (FIJI). Thresholding was performed to select for particles within 1–10 μm to eliminate clumps and background noise, and particle number was measured.

Analysis of apical explant staining and mitotic cell staging was performed at E14.5 in a 200 μm^2^ section of the apical surface. PH3 staining intensity varies by mitotic phase and was therefore used as a guide to indicate mitotic cells. Cells in prophase possess bright PH3 labelling with round DNA, metaphase cells possess PH3 labelling with DNA aligned at the metaphase plate, and ana/telophase cells rarely label with PH3 and display DNA separating in two aligned regions. All cells within the measurement area were examined carefully, and all mitotic cells were counted regardless of PH3 intensity.

At P21, cells were counted within a 400 μm width of cortex, or within a 1,000 μm width of the CA1/CA2 region of the hippocampus, counting all positive cells within the entire height of the cortex.

### 2.7 Statistical analyses

For each analysis, between three and eleven animals for each genotype group were assessed. Statistical significance was assessed using a one-way ANOVA and then a *post hoc* Tukey test, or a two-tailed Student’s t-test using GraphPad Prism (version 9.1.0 for Windows, GraphPad Software, San Diego, CA).

## 3 Results

### 3.1 Developmental loss of BubR1 causes mitotic defects in cortical neural progenitors

**FIGURE 1:**
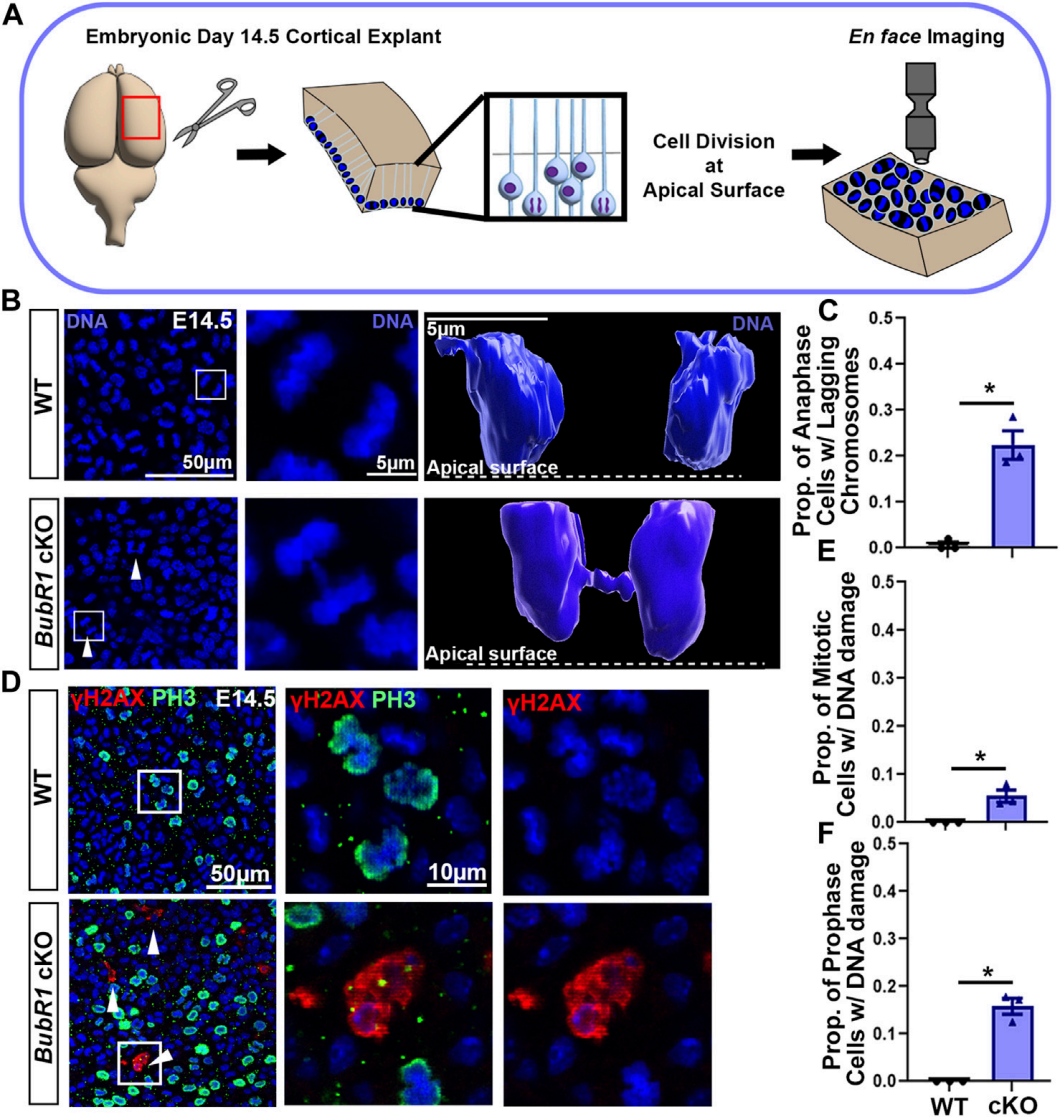
Loss of BubR1 causes lagging chromosomes in mitotic neural progenitors. **(A)** Schematic depicting the method by which dividing neural progenitors at the apical surface of the cortex can be visualized by *en face* apical explants. **(B, C)** Representative images and quantification showing an increase in the number of neural progenitors in cytokinesis that display lagging chromosomes in the BubR1 cKO brain. White arrows indicate cytokinetic cells with lagging chromosomes. White boxes indicate cells represented in inset images **(B)**. Scale bar = 50 μm. WT n = 3, *BubR1* cKO n = 3. (P = <0.0161). **(D–F)** Visualization and quantification of mitotic cells displaying DNA damage performed via apical explant staining shows a significant increase in mitotic cells with DNA damage in *BubR1* cKO brains. Cells with DNA damage are restricted to prophase where an average of 15% of prophase cells display DNA damage in the *BubR1* cKO. Scale bar = 50 μm, 10 μm. WT n = 3, *BubR1* cKO n = 3. (P^PH3+DNA DAMAGE^ = <0.0486), (P^PH3+DNA DAMAGE PROPHASE^ = .01100). Data are presented as the mean±SEM and analyzed by a two-tailed Student’s t-test.

As a major component of the spindle assembly check point (SAC), BubR1 is a versatile protein essential for genomic integrity. In addition to its role in delaying anaphase initiation by inhibiting the anaphase promoting complex (APC) regulatory subunit CDC20, BubR1 is known to stabilize microtubule-kinetochore attachment by recruiting phosphatase PP2A-B56 to reverse phosphorylation at kinetochores (Bolanos-Garcia and Blundell, 2011; Braga et al., 2020). This promotes chromosome alignment and SAC silencing. Although we have shown that BubR1 loss in cortical progenitors mediated by *Emx1-Cre* causes massive cell death and cortical cell reduction, the cellular mechanism responsible for compromised cellular viability is unknown (Simmons et al., 2019). Previously, a reduced proportion of mitotic cells and smaller fraction of metaphase cells in *BubR1* cKO cortices indicated the possibility of premature chromosome separation (Simmons et al., 2019). However, it was not determined whether mitotic disruption was present, or if BubR1-deficient mitosis generated aneuploidy or structural chromosome abnormalities. To find evidence of mitotic disruption, we performed *en face* apical explant analyses of *BubR1* cKO cortices with *Emx1-Cre* which we previously studied (Simmons et al., 2019). As cortical progenitors undergo cell cycle-dependent nuclear migration, mitotic division occurs at the ventricular (apical) surface (Figure 1A). Therefore, the apical surface of the developing cortex is populated by cells undergoing mitosis in both wild type (WT) and *BubR1* cKO animals (Figure 1A).

Through detailed analysis of mitotic cells at the height of neurogenesis in the embryonic day (E)14.5 cortex, we observed lagging chromosomes, DNA bridges, and micronuclei in *BubR1* cKO cells (Figure 1B and data not shown). Chromosome segregation defects are visible during anaphase and are known to contribute to the mosaic variegated aneuploidy that is associated with *BubR1* mutation or loss in humans. We found chromosome segregation defects in an average of 23% of total mitotic cells in *BubR1* cKO samples, compared to 0% of WT cells (Figures 1B, C). Modelling examples of anaphase cells using Imaris software allowed visualization of the distinct separation of anaphase DNA in WT cells, whereas a BubR1-deficient cell displayed a DNA bridge formed from lagging chromosomes lingering between separating DNA during anaphase (Figure 1B). Our observations of lagging chromosomes, DNA bridges, and micronuclei demonstrate a spectrum of chromosome segregation defects in BubR1-deficient neural progenitors. These results suggest that BubR1 loss contributes to chromosome segregation defects in cortical neural progenitors and may produce cells unfit for proliferation and survival.

Because aneuploidy can cause DNA damage that leads to cell cycle arrest and cell death, and we observed chromosome segregation defects, we next examined whether DNA damage is increased in *BubR1* cKO cortices. We were particularly interested in determining if mitotic cells harbor DNA damage as these cells that have passed the G2-M checkpoint could undergo mitotic catastrophe followed by apoptosis or necrosis (Yasuhira et al., 2016; Bertheloot et al., 2021). To determine the fraction of mitotic cells with DNA damage, we performed immunostaining on apical explants to co-label mitotic cells with phospho-histone (PH)3 and cells containing DNA double strand breaks with γH2AX. When BubR1 is lost during cortical development, we observed a significant increase in the number of cells displaying γH2AX^+^ DNA damage compared to WT controls (Figures 1D–F). Interestingly, mitotic cells with DNA damage were in prophase but not in later mitotic stages such as meta-, ana-, or telophase. Importantly, 10% of prophase cells have apparent DNA damage labelled by γH2AX in the *BubR1* cKO cortex while no WT cells in prophase are positive for γH2AX (Figure 1F). Prophase neural progenitors with DNA damage may struggle to enter subsequent mitotic phases. Consistent with this evidence, we previously observed that the proportion of mitotic cells in prophase was increased by *BubR1* deletion (Simmons et al., 2019). It is therefore possible that mitosis may be longer to provide time for the DNA damage response. Alternatively, cells with unresolved DNA damage may undergo mitotic catastrophe and cell death without proceeding through the rest of the mitotic cycle. Together, we have found that in the absence of BubR1, a significant proportion of prophase mitotic cells contain DNA damage.

### 3.2 Apoptotic cell death but not DNA damage can be improved by *Trp53* co-deletion

**FIGURE 2:**
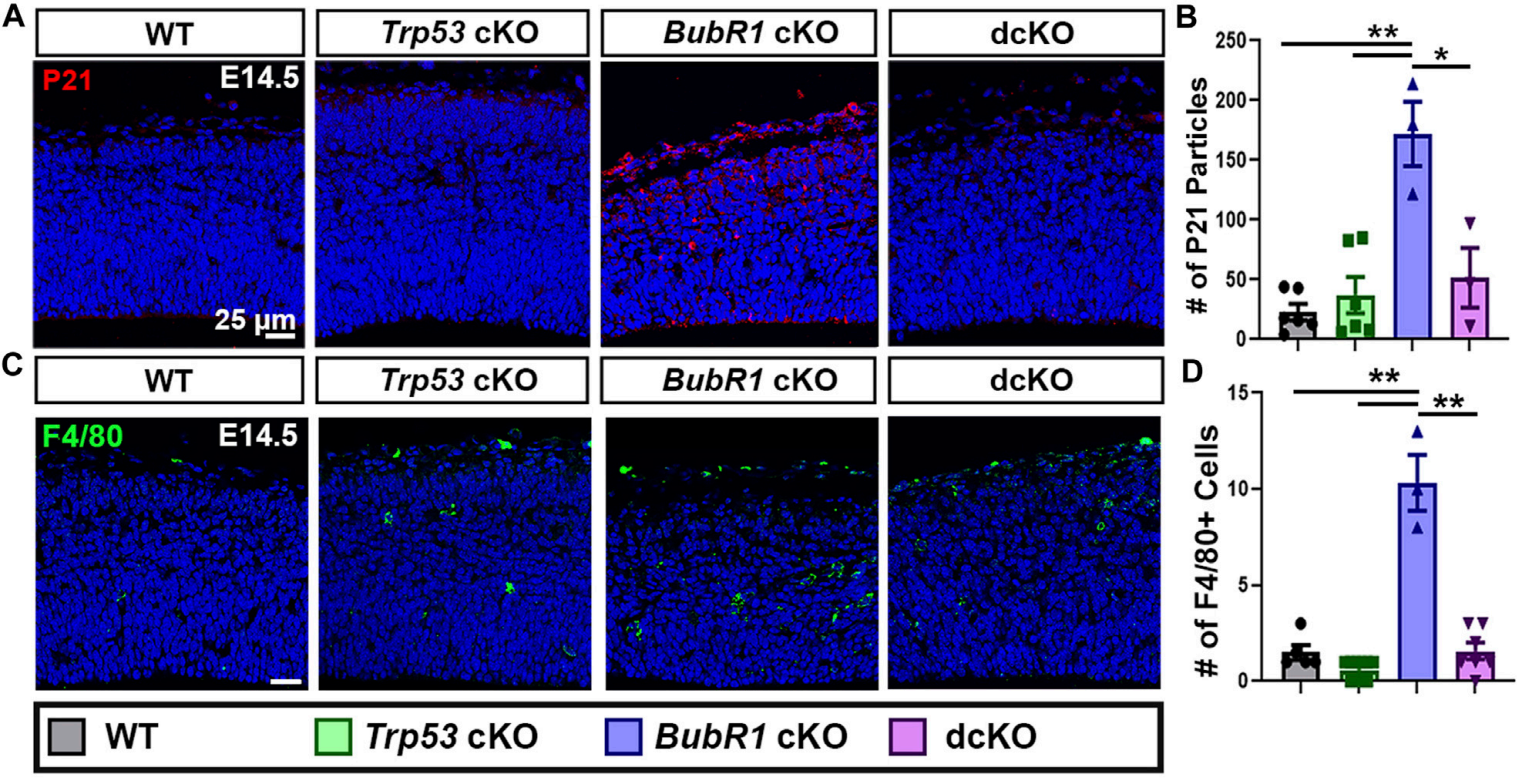
P53 activation occurs in response to BubR1 loss. **(A, B)** At E14.5, immunohistochemistry, and particle analysis for P21 in a 200 μm width of the cortex demonstrates that *BubR1* cKO animals express more P21 protein. This effect can be fully rescued by Trp53 deletion indicating that P53 is activated in response to BubR1 loss. Scale bar = 25 μm. WT n = 6, *P53* cKO n = 6, *BubR1* cKO n = 3, dcKO n = 3. (ANOVA^P21^ F = 13.97, df = 3.14, *p* = 0.0002; Adjusted P^WT-p53 CKO^ = 0.8886, Adjusted P^WT−BUBR1 CKO^ = 0.0001, Adjusted P^WT−DCKO^ = 0.6455, Adjusted P^P53 CKO−BUBR1 cKO^ = 0.0004, Adjusted P^P53 CKO−DCKO^ = 0.9305, Adjusted P^BUBR1−DCKO^ = 0.0036). **(C, D)** Representative images and quantification of F4/80 labeled activated microglia. Activated microglia are increased in the cortex when BubR1 is lost. This effect is returned to WT levels in the dcKO group, confirming P53 activation when BubR1 is lost. Scale bar = 25 μm. WT n = 5, *P53* cKO n = 10, *BubR1* cKO n = 3, dcKO n = 7. (ANOVA^F4/80^ F = 62.25, df = 3.21, P = <0.0001; Adjusted P^WT-p53 CKO^ = 0.4648, Adjusted P^WT−BUBR1 CKO^ = <0.0001, Adjusted P^WT−DCKO^ = 0.9995, Adjusted P^P53 CKO−BUBR1 cKO^ = <0.0001, Adjusted P^P53 CKO−DCKO^ = 0.3107, Adjusted P^BUBR1−DCKO^ = <0.0001).

Because significant cell death and DNA damage are features of BubR1 cortical loss, we examined whether P53 activation mediates cell elimination in the *BubR1* cKO cortex as in many other microcephaly models. To determine whether P53 is indeed activated in response to BubR1 loss in the developing cortex, we performed immunostaining for P21, the well-known downstream effector of P53. Importantly, P21 levels were significantly increased throughout the *BubR1* cKO cortex at E14.5 (Figures 2A, B). To be certain that P53 activation causes P21 upregulation, we genetically deleted *Trp53* in our *BubR1* cKO using *Emx1-Cre.* As expected, *Trp53* co-deletion reduced P21 expression to WT levels (Figures 2A, B). Because microglia are known to become activated in the cortex following P53 upregulation, we also analyzed the numbers of activated microglia (F4/80^+^) in the developing cortex (Shi et al., 2019). The numbers of activated microglia were significantly increased in the *BubR1* cKO and reduced back to WT levels by *Trp53* co-deletion (Figures 2C, D). Together, this data indicates that P53 is activated when BubR1 is lost from the developing cortex.

**FIGURE 3:**
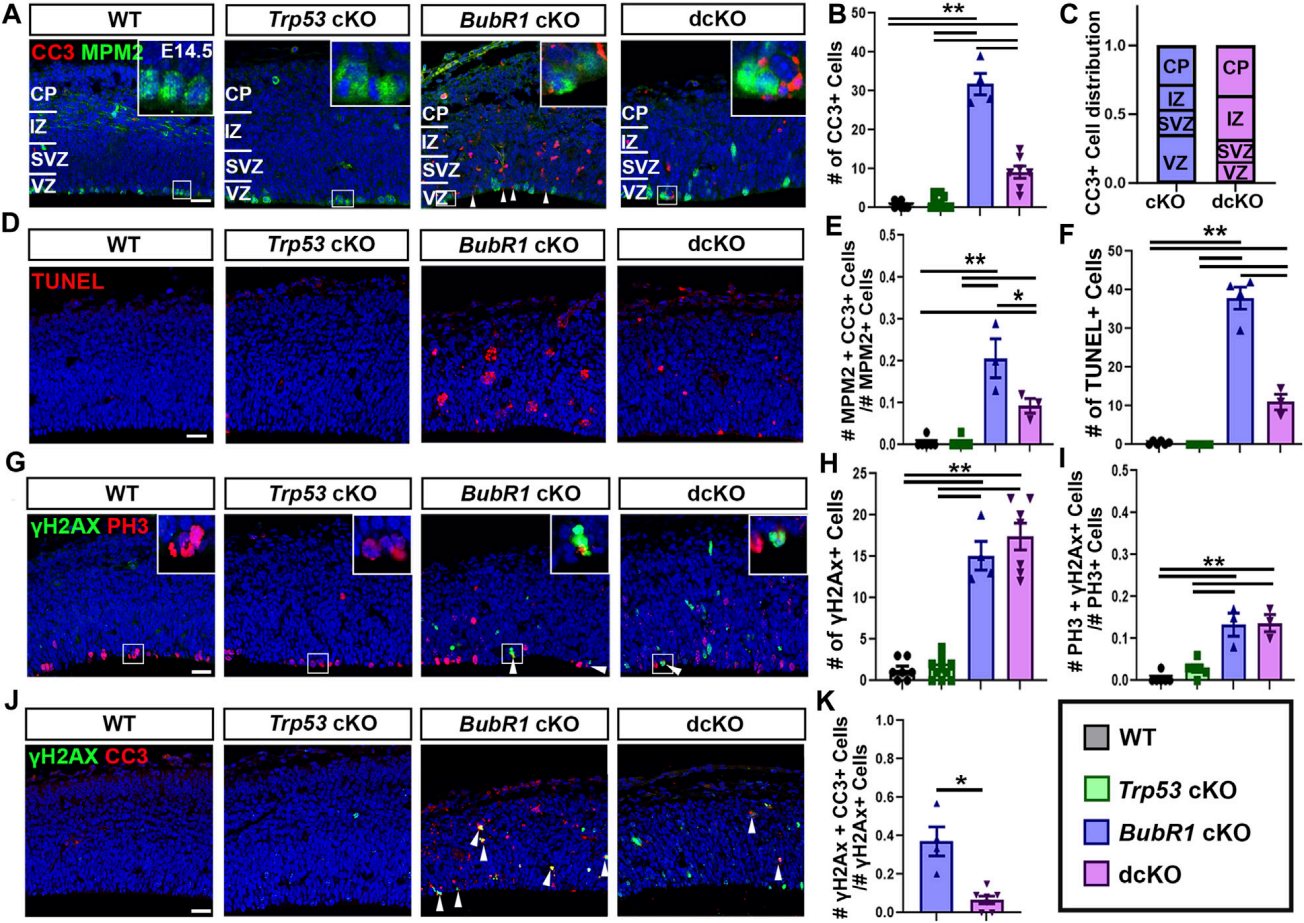
At E14.5, microcephaly pathology can be partially improved by *Trp53* co-deletion. **(A)** Representative images showing CC3 labeling of apoptotic cells and MPM2 labelling of mitotic cells in the E14.5 cortex. **(B)** Quantification of CC3^+^ apoptotic cells in a 200 μm width of the cortex demonstrates a significant increase in CC3^+^ cells in *BubR1* cKO animals. Cell death still occurs but is significantly reduced in dcKO brains. Scale bar = 25 μm. WT n = 7, *P53* cKO n = 10, *BubR1* cKO n = 4, dcKO n = 7. (ANOVA^CC3^ F = 107.8, df = 3.24, P = <0.0001; Adjusted P^WT-p53 CKO^ = 0.9686, Adjusted P^WT−BUBR1 CKO^ = <0.0001, Adjusted P^WT−DCKO^ = 0.0002, Adjusted P^P53 CKO−BUBR1 cKO^ = <0.0001, Adjusted P^P53 CKO−DCKO^ = 0.0002, Adjusted P^BUBR1−DCKO^ = <0.0001). **(C)** Quantification of the distribution of CC3^+^ dying cells throughout the cortex showing that cell death affects cells in all cortical regions. The proportion of dying cells is not significantly different between any region or between the two animal groups. *BubR1* cKO n = 3, dcKO n = 3. (ANOVA^CC3/Cortical Zone^ (Row^CKO/DCKO^: DF=1, F=<.0001, P=>.99)(Column^Region^: DF=3, F=.6967, P=.6175). **(D)** Representative images of TUNEL^+^ cells. Scale bar = 25 μm. **(E)** Quantification of the proportion of mitotic cells undergoing cell death in a 200 μm width of the cortex at E14.5. The proportion of dying mitotic cells is significantly higher in *BubR1* cKO animals compared to WT or P53 cKO. This effect is significantly but not fully reduced in the dcKO group. WT n = 6, P53 cKO n = 6, BubR1 cKO n = 3, dcKO n = 3. (ANOVA^MPM2/CC3^ F = 29.72, df = 3.14, P = <0.0001; Adjusted P^WT-p53 CKO^ = >0.9999, Adjusted P^WT–BUBR1 CKO^ = <0.0001, Adjusted P^WT–DCKO^ = 0.0120, Adjusted P^P53 CKO–BUBR1 cKO^ = <0.0001, Adjusted P^P53 CKO–DCKO^ = 0.0120, Adjusted P^BUBR1–DCKO^ = 0.0055). **(F)** Quantification of of TUNEL^+^ cells in a 200 μm width of the cortex demonstrates a significant increase in TUNEL^+^ cells in *BubR1* cKO animals. Cell death still occurs but is significantly reduced in dcKO brains. WT n = 6, P53 cKO n = 6, *BubR1* cKO n = 4, dcKO n = 3. (ANOVA^TUNEL^ F = 172.0, df = 3.15, P = <0.0001; Adjusted P^WT-p53 CKO^ = 0.9942, Adjusted P^WT–BUBR1 CKO^ = <0.0001, Adjusted P^WT–DCKO^ = 0.0006, Adjusted P^P53 CKO–BUBR1 cKO^ = <0.0001, Adjusted P^P53 CKO–DCKO^ = 0.0004, Adjusted P^BUBR1–DCKO^ = <0.0001). **(G–I)** Representative images and quantification of mitotic cells and DNA damage in a 200 μm width of the cortex. Quantification of cells with DNA damage (γH2AX^+^) at E14.5 in a 200 μm width of the cortex reveals that the number of cells with DNA image is increased in *BubR1* cKO but is not improved by *Trp53* co-deletion. Scale bar = 25 μm. WT n = 7, *P53* cKO n = 10, *BubR1* cKO n = 3, dcKO n = 7. (ANOVA^γH2AX^ F = 71.37, df = 3.24, P = <0.0001; Adjusted P^WT-p53 CKO^ = 0.9985, Adjusted P^WT−BUBR1 CKO^ = <0.0001, Adjusted P^WT−DCKO^ = <0.0001, Adjusted P^P53 CKO−BUBR1 cKO^ = <0.0001, Adjusted P^P53 CKO−DCKO^ = <0.0001, Adjusted P^BUBR1−DCKO^ = 0.5170). In BubR1 cKO animals, there is a significant increase in mitotic cells with DNA damage compared to WT that is unchanged by Trp53 deletion. Scale bar = 25 μm. WT n = 6, *P53* cKO n = 6, *BubR1* cKO n = 3, dcKO n = 3. (ANOVA^γH2AX/PH3^ F = 28.13, df = 3.14, P = <0.0001; Adjusted P^WT-p53 CKO^ = 0.4383, Adjusted P^WT−BUBR1 CKO^ = <0.0001, Adjusted P^WT−DCKO^ = <0.0001, Adjusted P^P53 CKO−BUBR1 cKO^ = 0.0003, Adjusted P^P53 CKO−DCKO^ = 0.0002, Adjusted P^BUBR1−DCKO^ = 0.9986). **(J–K)** Quantification of the proportion of γH2AX^+^ cells with DNA damage that are undergoing apoptosis (CC3^+^) shows that a significant number of cells with DNA damage are dying in the *BubR1* cKO but not the dcKO animals. Scale bar = 25 μm *BubR1* cKO n = 4, dcKO n = 7. (*p* = .0239). White boxes represent inset images. Inset images are closer representations of mitotic cells. Data are presented as the mean±SEM and analyzed by one-way ANOVA followed by a posthoc Tukey test, two-way ANOVA, or a Student’s t-test.

Next, we sought to determine the contribution of P53 activation to cell death when BubR1 is lost via the presence of the apoptosis marker cleaved-caspase 3 (CC3) in WT, *Trp53* cKO, *BubR1* cKO and dcKO animals at E14.5. Because P53 is a major apoptotic cell death mediator, we observed an expected, nearly complete absence of CC3^+^ cells in *Trp53* cKO cortices (Figures 3A, B). In contrast, BubR1 loss results in a significant increase in the number of apoptotic cortical cells (Figures 3A, B). To determine whether P53 activation is responsible for apoptotic cell death, we analyzed CC3^+^ cells in dcKO samples. Interestingly, *Trp53* co-deletion produced a significant but incomplete reduction in apoptotic cell numbers (Figures 3A, B). To better characterize the population of cortical cells being affected by cell death, we divided each cortex into four regions (Figure 3A). These regions include the ventricular zone (VZ), subventricular zone (SVZ), intermediate zone (IZ), and cortical plate (CP). Our analysis revealed that cell death is distributed throughout the cortex. Cell death was not significantly different between regions of the cortex or between the cKO and dcKO groups (Figure 3C). This data therefore suggests that cortical cells residing in any region can be affected by cell death when BubR1 is lost in the developing cortex. This data reflects previous analysis of cell death in *BubR1* cKO cortices which showed that both proliferative and post-mitotic cells undergo cell death when BubR1 is lost (Simmons et al., 2019).

To confirm that *Trp53* deletion does not fully rescue cell death in the *BubR1* cKO cortex, we also performed TUNEL staining to label cells in the terminal stages of apoptosis. While WT or *Trp53* cKO cortices include very few TUNEL^+^ cells, deletion of *BubR1* significantly increases dying cells, and *Trp53* co-deletion significantly but not fully reduces the average number of TUNEL^+^ cells (Figures 3D, F). Importantly, we also found a substantial proportion of mitotic cells undergoing apoptosis (∼20%) in the *BubR1* cKO cortex, which can be halved by *Trp53* co-deletion (10%) (Figure 3E). Because *Trp53* deletion does not fully prevent apoptotic cell death, as indicated by CC3 staining and TUNEL labeling, our results suggest the presence of a P53-independent cell death mechanism affecting BubR1-deficient cortical cells.

Because P53 activation can cause DNA damage and DNA damage can cause P53 activation, we asked whether DNA damage persists after *Trp53* deletion. If P53 activation contributes to DNA damage accumulation, DNA damage would be reduced by P53 depletion. Alternatively, P53 depletion could allow the persistence of cells with DNA damage by preventing them from being repaired or eliminated. As previously shown (Simmons et al., 2019), we observed that the number of cells containing γH2AX^+^ DNA damage in the cortex was significantly increased by loss of BubR1 (Figures 3G, H). However, *Trp53* co-deletion did not decrease the number of cells with DNA damage, suggesting that P53 activation is downstream of or unrelated to DNA damage acquisition (Figure 3H). Similarly, an increase in the proportion of mitotic cells with DNA damage (13%) was unchanged by *Trp53* co-deletion (Figure 3I). This data suggests that P53-mediated damage repair does not play a significant role when BubR1 is lost because elimination of P53 in the dcKO cortex does not increase the number of cells with DNA damage compared to *BubR1* cKO brains.

Although cells with unrepaired DNA damage should undergo apoptosis to prevent genomic instability, the absence of P53 in dcKO cortices may prevent apoptosis of genomically unfit cells. To test this, we analysed the proportion of cells with DNA damage (γH2AX^+^) that were undergoing apoptotic cell death (CC3^+^) (Figures 3J, K). Due to their lack of significant cell death or DNA damage, WT and *Trp53* cKO animal groups were excluded from this analysis. We found that in the *BubR1* cKO cortex, an average of 37% of cells with γH2AX^+^ DNA damage were CC3^+^ apoptotic cells (Figure 3K). Meanwhile, dcKO cortices showed that only 7% of cells with DNA damage are apoptotic (Figure 3K), suggesting that cells with DNA damage are no longer targeted for apoptosis in the absence of P53. Together, our results suggest that apoptosis triggered by DNA damage is significantly reduced when P53 is eliminated, potentially allowing cells with DNA damage to persist in dcKO cortices at this stage.

### 3.3 *Trp53* co-deletion partially restores cortical cells during neurogenesis

**FIGURE 4:**
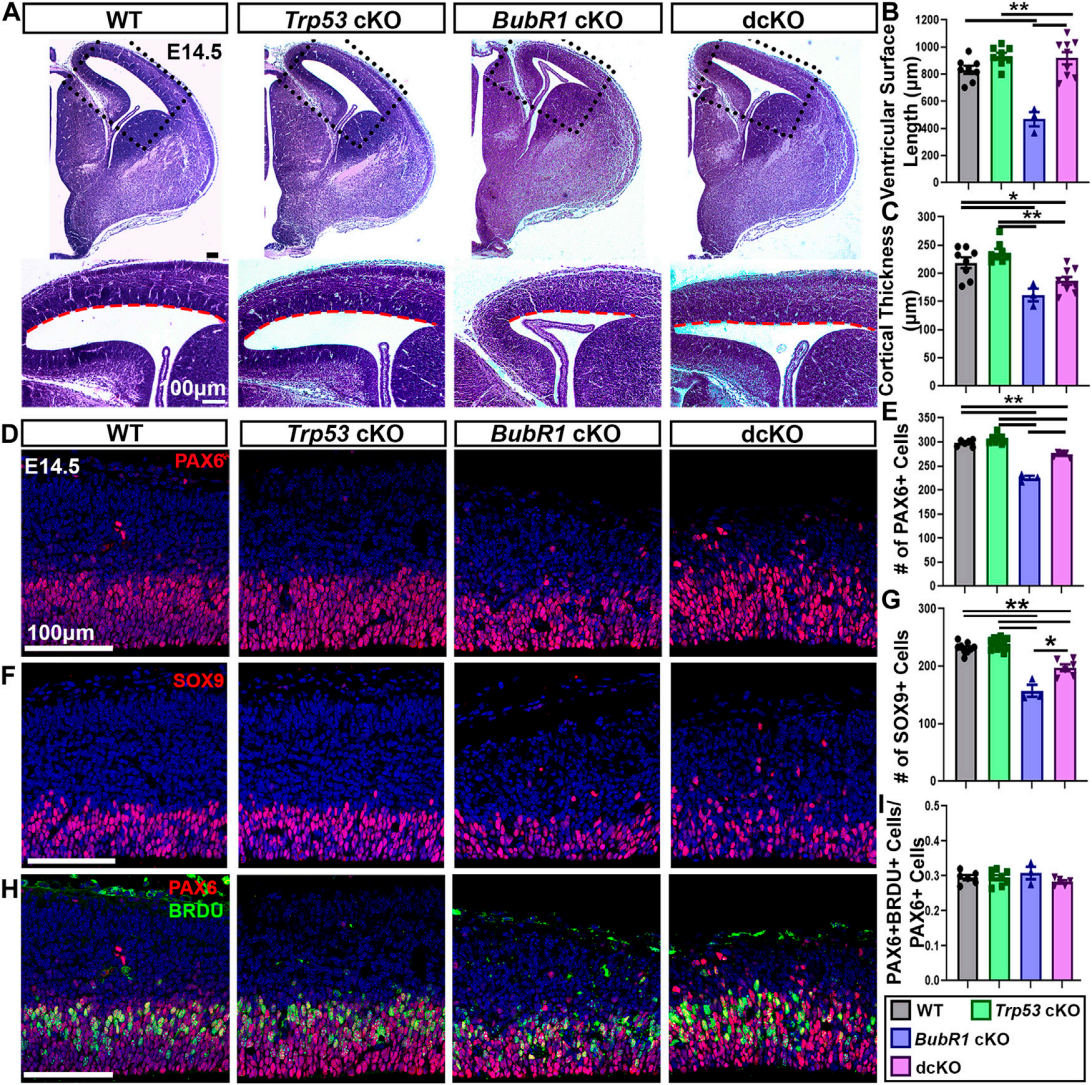
At E14.5, microcephaly pathology can be partially improved by *Trp53* co-deletion. **(A)** At E14.5 Hematoxylin and Eosin staining demonstrates that ventricular surface length, but not cortical thickness is improved in dcKO cortices compared to *BubR1* cKO mice that display a reduced cortex. Scale bar = 100 μm. **(B, C)** Quantification of ventricular surface length and cortical thickness at E14.5. WT n = 8, *P53* cKO n = 8, *BubR1* cKO n = 3, dcKO n = 9. (ANOVA^LENGTH^ F = 17.29, df = 3.24, P = <0.0001; Adjusted P^WT-p53 CKO^ = 0.2214, Adjusted P^WT−BUBR1 CKO^ = 0.0001, Adjusted P^WT−DCKO^ = 0.3308, Adjusted P^P53 CKO−BUBR1 cKO^ = <0.0001, Adjusted P^P53 CKO−DCKO^ = 0.9892, Adjusted P^BUBR1−DCKO^ = <0.0001). (ANOVA^THICKNESS^ F = 12.38, df = 3.24, P = <0.0001; Adjusted P^WT-p53 CKO^ = 0.3791, Adjusted P^WT−BUBR1 CKO^ = 0.0043, Adjusted P^WT−DCKO^ = 0.0299, Adjusted P^P53 CKO−BUBR1 cKO^ = 0.0002, Adjusted P^P53 CKO−DCKO^ = 0.0005, Adjusted P^BUBR1−DCKO^ = 0.3470). **(D, E)** Representative images and quantification of PAX6 labeling of neural progenitors at E14.5 in a 200 μm width of the cortex. PAX6^+^ cell numbers are reduced by BubR1 loss and significantly but not fully rescued in the dcKO. Scale bar = 100 μm. WT n = 6, *P53* cKO n = 8, *BubR1* cKO n = 3, dcKO n = 5. (ANOVA^PAX6^ F = 91.02, df = 3.18, P = <0.0001; Adjusted P^WT-p53 CKO^ = 0.2309, Adjusted P^WT−BUBR1 CKO^ = <0.0001, Adjusted P^WT−DCKO^ = 0.0003, Adjusted P^P53 CKO−BUBR1 cKO^ = <0.0001, Adjusted P^P53 CKO−DCKO^ = <0.0001, Adjusted P^BUBR1−DCKO^ = <0.0001). **(F, G)** Representative images and quantification of SOX9^+^ neural progenitors in a 200 μm width of cortex at E14.5 shows that cell numbers are significantly reduced by BubR1 loss and are not rescued by subsequent *Trp53* co-deletion. Scale bar = 100 μm. WT n = 9, *P53* cKO n = 11, *BubR1* cKO n = 3, dcKO n = 6. (ANOVA^SOX9^ F = 47.85, df = 3.25, P = <0.0001; Adjusted P^WT-p53 CKO^ = 0.5383, Adjusted P^WT−BUBR1 CKO^ = <0.0001, Adjusted P^WT−DCKO^ = <0.0001, Adjusted P^P53 CKO−BUBR1 cKO^ = <0.0001, Adjusted P^P53 CKO−DCKO^ = <0.0001, Adjusted P^BUBR1−DCKO^ = 0.0003). **(H, I)** At E14.5, representative images and quantification of the proportion of PAX6^+^ neural progenitors that are also BrdU^+^ after 30 min of BrdU incorporation *in utero* shows that the proportion of S-phase neural progenitors is unchanged by loss of BubR1 or P53. Scale bar = 100 μm. WT n = 6, *P53* cKO n = 8, *BubR1* cKO n = 3, dcKO n = 5. (ANOVA^PAX6/BrdU^ F = 1.070, df = 3.18, *p* = 0.3866; Adjusted P^WT-p53 CKO^ = >0.9999, Adjusted P^WT−BUBR1 CKO^ = 0.7737, Adjusted P^WT−DCKO^ = 0.7432, Adjusted P^P53 CKO−BUBR1 cKO^ = 0.7398, Adjusted P^P53 CKO−DCKO^ = 0.7199, Adjusted P^BUBR1−DCKO^ = 0.3191). Data are presented as the mean±SEM and analyzed by one-way ANOVA followed by a posthoc Tukey test.

Since we found substantial reductions in apoptotic but not DNA damaged cells in dcKO cortices, we next sought to determine if *Trp53* deletion could improve cell survival in the developing cortex. As previously shown, at E14.5 BubR1 loss results in significantly reduced ventricular length and cortical thickness, indicating a smaller dorsal cortex (Figures 4A–C). While *Trp53* co-deletion significantly improved ventricular surface length to WT levels, there was no improvement in cortical thickness (Figures 4A–C). This suggests that initial microcephaly pathogenesis is not completely rescued by eliminating P53-dependent apoptotic cell death at E14.5. To determine if the lack of rescue in cortical thickness correlated with cell numbers, we first labeled and analyzed the number of apical radial glia progenitors (aRG, PAX6^+^). Consistent with previous results, we found a significant reduction in aRG numbers caused by BubR1 loss (Figures 4D, E). Subsequent co-deletion of *Trp53* partially rescued the number of PAX6^+^ neural progenitors (Figures 4D, E). As in our analysis of PAX6^+^ aRG, we found that SOX9^+^ aRG were reduced by the loss of BubR1 and were partially rescued by *Trp53* co-deletion (Figures 4F, G).

Although we did not previously find changes in the S-phase fraction in *BubR1* cKO cortices, P53 has established functions in cell cycle arrest and delayed mitotic progression (Ohashi et al., 2015; Pilaz et al., 2016; Soto et al., 2017; Hafner et al., 2019; Navarro et al., 2020). Therefore, we determined whether the S-phase fraction is altered when P53 is lost. To determine if the proportion of S-phase aRG was altered by BubR1 loss or *Trp53* co-deletion, we used BrdU and Pax6 immunostaining after treating mice with nucleotide analog BrdU for 30 min. This analysis revealed no significant differences in the proportion of S-phase cells among total aRG in any groups (Figures 4H, I). Therefore, it is unlikely that cell cycle progression is affected by BubR1 or P53 depletion.

**FIGURE 5:**
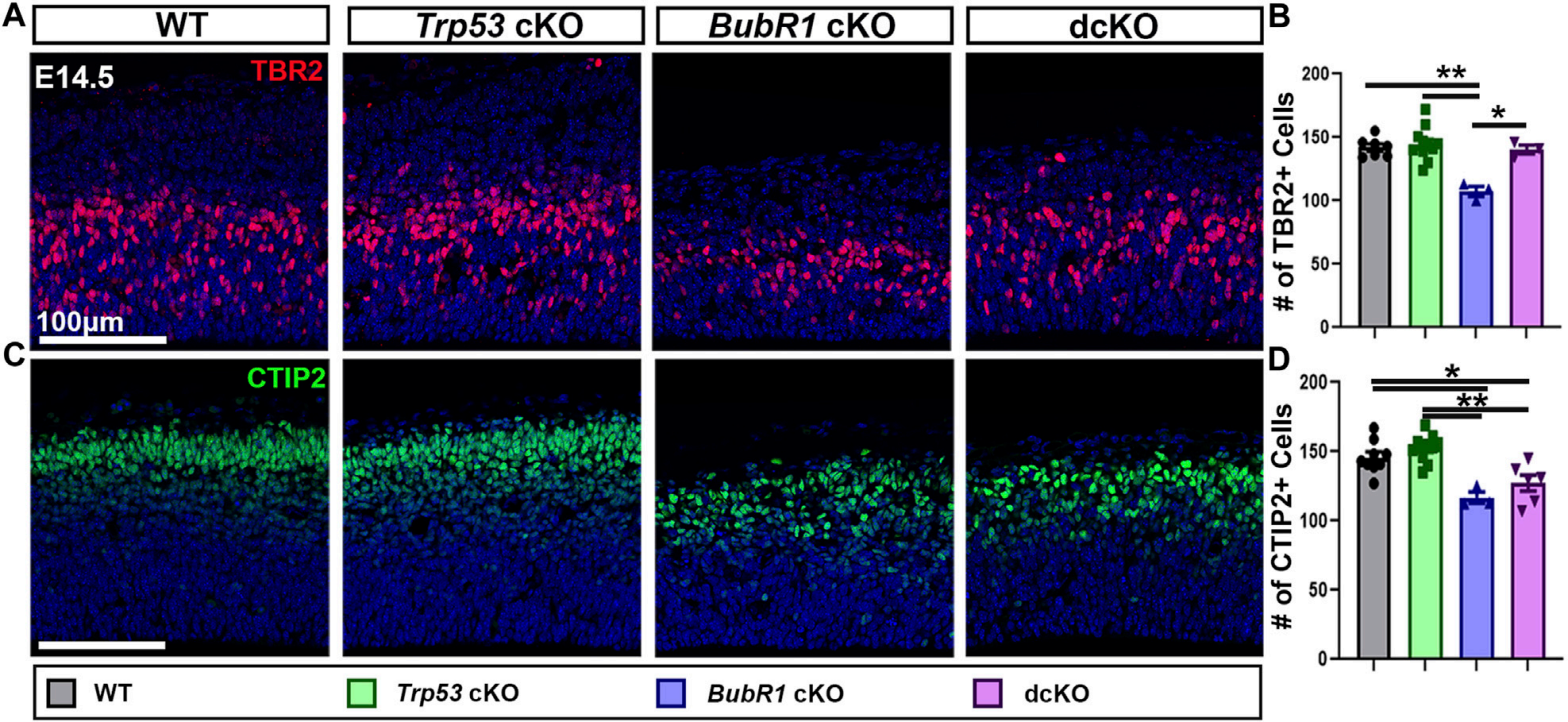
Intermediate progenitor and neuron numbers are partially rescued by *Trp53* co-deletion at E14.5. **(A, B)** Representative images and quantification of TBR2 labeling of intermediate progenitors in a 200 μm width of the cortex at E14.5. TBR2^+^ cell numbers are fully rescued in the dcKO group from their reduced levels in *BubR1* cKO cortices. Scale bar = 100 μm. WT n = 7, *P53* cKO n = 11, *BubR1* cKO n = 3, dcKO n = 3. (ANOVA^TBR2^ F = 10.21, df = 3.20, *p* = 0.0003; Adjusted P^WT-p53 CKO^ = 0.9522, Adjusted P^WT−BUBR1 CKO^ = 0.0007, Adjusted P^WT−DCKO^ = 0.9957, Adjusted P^P53 CKO−BUBR1 cKO^ = 0.0001, Adjusted P^P53 CKO−DCKO^ = 0.9209, Adjusted P^BUBR1−DCKO^ = 0.0054). **(C, D)** Representative images and quantification of CTIP2-labeled early-born neurons in a 200 μm width of the cortex at E14.5. The reduction in numbers observed in the *BubR1* cKO animals is unaltered in the dcKO. Scale bar = 100 μm. WT n = 9, *P53* cKO n = 11, *BubR1* cKO n = 3, dcKO n = 6. (ANOVA^CTIP2^ F = 12.17, df = 3.25, P = <0.0001; Adjusted P^WT-p53 CKO^ = 0.5639, Adjusted P^WT−BUBR1 CKO^ = 0.0032, Adjusted P^WT−DCKO^ = 0.0199, Adjusted P^P53 CKO−BUBR1 cKO^ = 0.0003, Adjusted P^P53 CKO−DCKO^ = 0.0008, Adjusted P^BUBR1−DCKO^ = 0.5507). Data are presented as the mean±SEM and analyzed by one-way ANOVA followed by a posthoc Tukey test.

Next, we examined the number of intermediate basal progenitors (BP, TBR2^+^), and early-born neurons (CTIP2^+^) (Figure 5). Similar to aRG, BP and early-born neuron numbers were reduced in *BubR1* cKO brains. Interestingly, while the number of TBR2^+^ BPs was fully rescued to WT levels in the dcKO cortex (Figures 5A, B), the number of CTIP2^+^ early-born neurons was not rescued at all by P53 removal (Figures 5C, D). These results demonstrate the differing effects of *Trp53* deletion on various cell types in the developing cortex. Interestingly, *Trp53* deletion improved ventricular surface length but not cortical thickness (Figures 4A–C). It is possible that because the ventricular surface is composed of apical endfeet and cells undergoing mitosis, the partial but significant restoration of PAX6^+^ cell numbers may contribute to the ventricular length rescue at E14.5. On the other hand, because vertical growth is achieved by increasing cortical plate thickness, the lack of rescue in cortical neuron production may explain why cortical thickness is not rescued in dcKO brains. Although the number of BPs are restored in dcKO cortices, the partial rescue of aRG numbers and failure to rescue early-born neuron production suggests that prevention of P53-dependent apoptotic cell death may have a limited ability to restore cortical cell numbers.

### 3.4 P53 removal does not rescue the microcephaly phenotype of *BubR1* cKO mice

**FIGURE 6:**
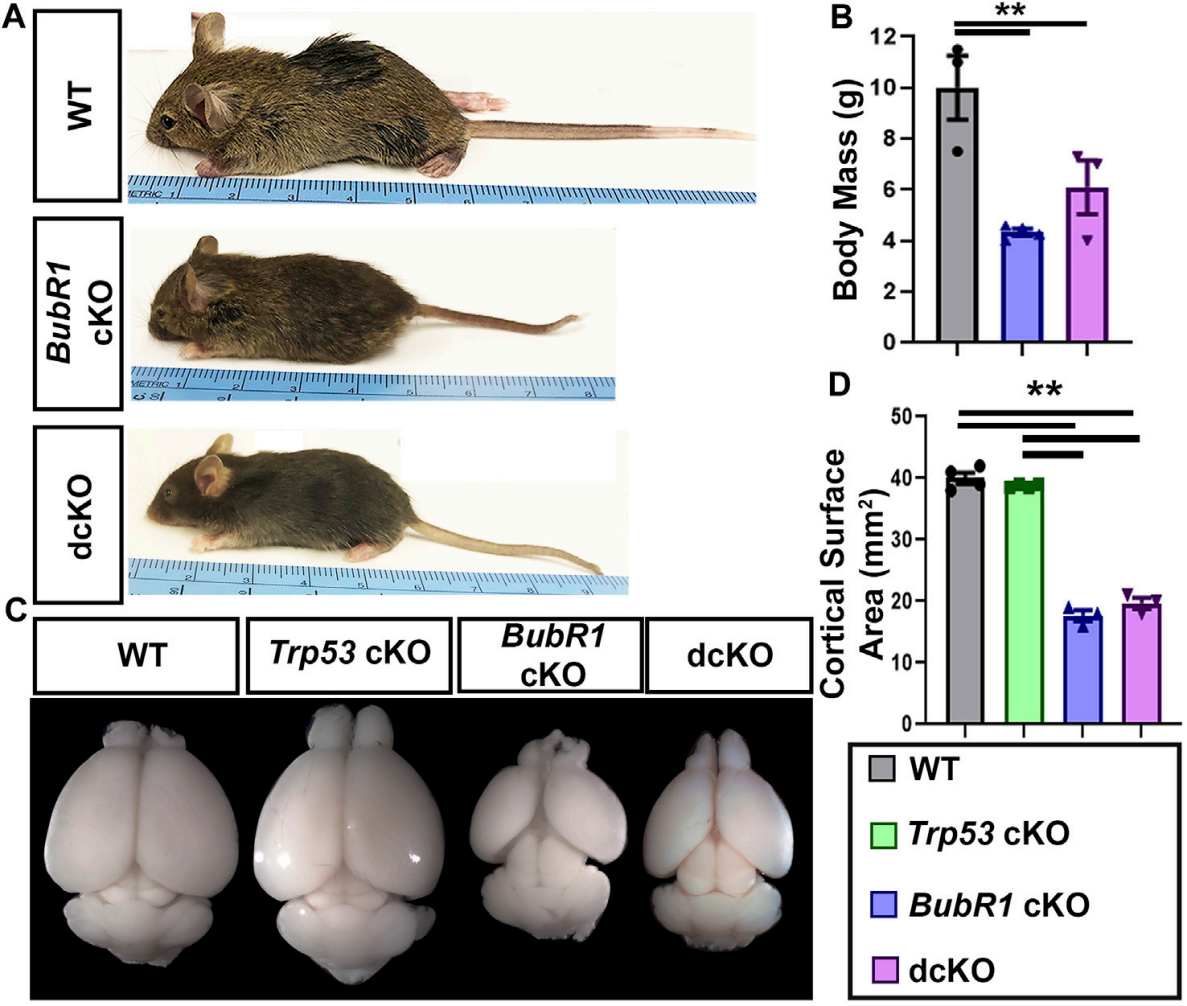
*BubR1 cKO* and dcKO show reductions in body and cortex size. **(A, B)** Representative images and quantification show that *BubR1* cKO and dcKO animals are smaller than their WT counterparts at P21. WT n = 3, *BubR1* cKO n = 4, dcKO n = 3. (ANOVA^BODY MASS^ F = 11.95, df = 2.7, *p* = 0.0055; Adjusted P^WT−BUBR1 CKO^ = 0.0046, Adjusted P^WT−DCKO^ = 0.0393, Adjusted P^BUBR1−DCKO^ = 0.3436). **(C, D)** Representative images of whole brains and quantification of cortical area at P21 shows an overall cortical reduction in *BubR1* cKO animals that is unchanged in dcKO animals. WT n = 4, *P53* cKO n = 4, *BubR1* cKO n = 3, dcKO n = 3. (ANOVA^CORTICAL AREA^ F = 251.8, df = 3.10, P = <0.0001; Adjusted P^WT-p53 CKO^ = 0.6042, Adjusted P^WT−BUBR1 CKO^ = <0.0001, Adjusted P^WT−DCKO^ = <0.0001, Adjusted P^P53 CKO−BUBR1 cKO^ = <0.0001, Adjusted P^P53 CKO−DCKO^ = <0.0001, Adjusted P^BUBR1−DCKO^ = 0.3496). Data are presented as the mean±SEM and analyzed by one-way ANOVA followed by a posthoc Tukey test.

**FIGURE 7:**
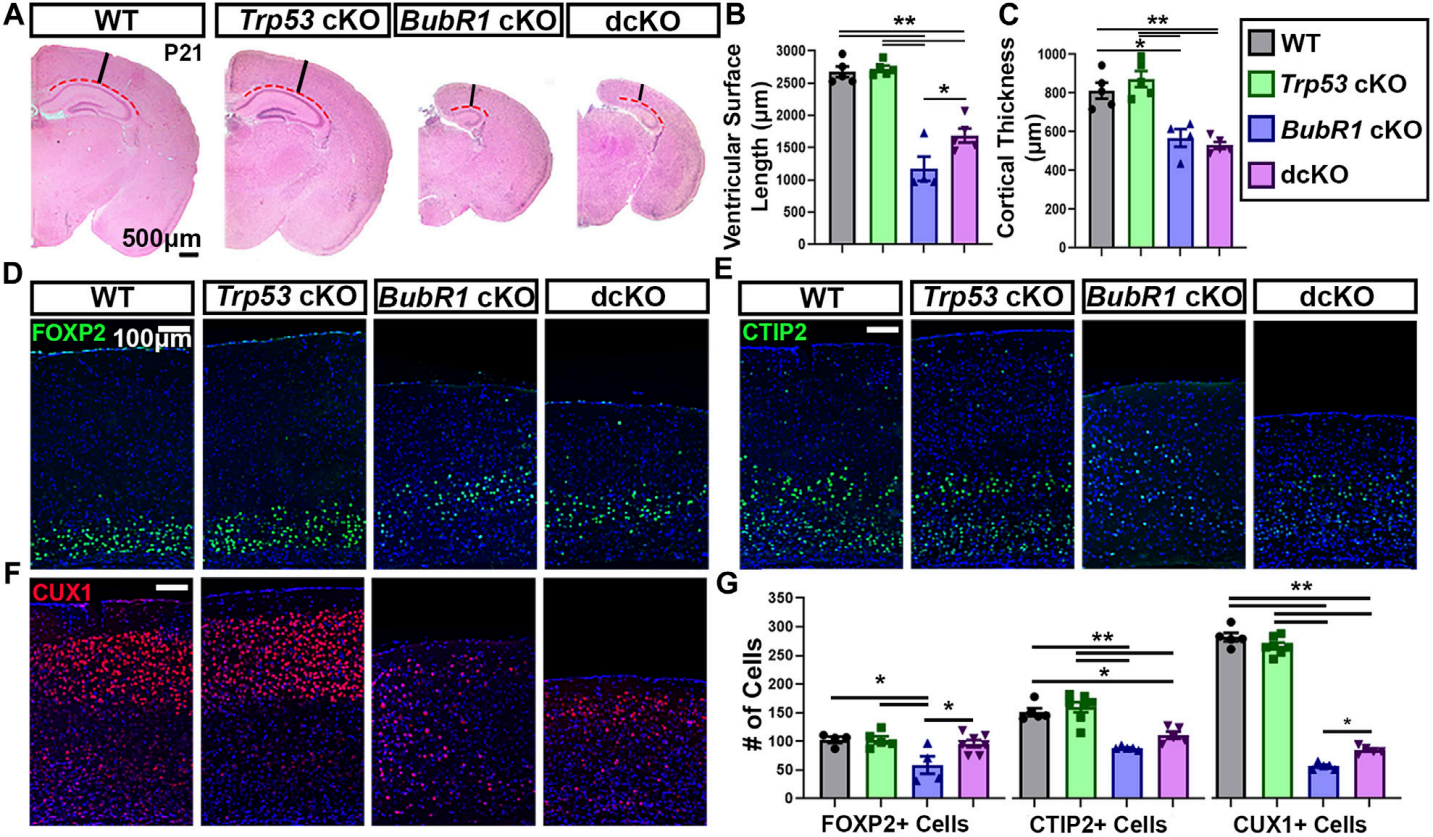
Concurrent *Trp53* deletion only partially rescues microcephaly pathology in *BubR1* cKO animals at P21. **(A)** Hematoxylin and Eosin staining shows that *BubR1* cKO causes microcephaly at P21 which is minimally improved in dcKO animals. Scale bar = 500 μm. Red dotted lines denote where the ventricular surface area was measured. Black lines indicate where cortical thickness was measured. **(B, C)** Quantification of ventricular surface length and cortical thickness at P21 indicates a mild rescue of ventricular length but not cortical thickness in the dcKO compared to *BubR1* cKO. WT n = 5, *P53* cKO n = 5, *BubR1* cKO n = 4, dcKO n = 5. (ANOVA ^LENGTH^ F = 47.50, df = 3.15, P = <0.0001; Adjusted P^WT-p53 CKO^ = 0.9870, Adjusted P^WT−BUBR1 CKO^ = <0.0001, Adjusted P^WT−DCKO^ = <0.0001, Adjusted P^P53 CKO−BUBR1 cKO^ = <0.0001, Adjusted P^P53 CKO−DCKO^ = <0.0001, Adjusted P^BUBR1−DCKO^ = 0.0246). (ANOVA ^THICKNESS^ F = 21.53, df = 3.15, P = <0.0001; Adjusted P^WT-p53 CKO^ = 0.6554, Adjusted P^WT−BUBR1 CKO^ = 0.0022, Adjusted P^WT−DCKO^ = 0.0003, Adjusted P^P53 CKO−BUBR1 cKO^ = 0.0003, Adjusted P^P53 CKO−DCKO^ = <0.0001, Adjusted P^BUBR1 DCKO^ = 0.8940).) **(D–F)** Immunohistochemistry for FOXP2, CTIP2, and CUX1 demonstrates that *BubR1* cKO causes a reduction in both early-born and late-born populations of neurons, while concurrent Trp53 deletion minimally improves cell loss. Scale bar = 100 μm. **(G)** Quantification of cortical neuron numbers at P21 in a 400 μm width of the cortex. (FOXP2: WT n = 4, *P53* cKO n = 5, *BubR1* cKO n = 4, dcKO n = 7. ANOVA^FOXP2^ F = 5.162, df = 3.16, *p* = 0.0875; Adjusted P^WT-p53 CKO^ = >0.9999, Adjusted P^WT−BUBR1 CKO^ = 0.0222, Adjusted P^WT−DCKO^ = 0.9455, Adjusted P^P53 CKO−BUBR1 cKO^ = 0.0154, Adjusted P^P53 CKO−DCKO^ = 0.9330, Adjusted P^BUBR1−DCKO^ = 0.0287). (CTIP2: WT n = 5, *P53* cKO n = 7, *BubR1* cKO n = 5, dcKO n = 5. ANOVA^CTIP2^ F = 22.34, df = 3.18, P = <0.0001; Adjusted P^WT-p53 CKO^ = 0.8262, Adjusted P^WT−BUBR1 CKO^ = <0.0001, Adjusted P^WT−DCKO^ = 0.0569, Adjusted P^P53 CKO−BUBR1 cKO^ = <0.0001, Adjusted P^P53 CKO−DCKO^ = 0.0006, Adjusted P^BUBR1−DCKO^ = 0.1781). (CUX1: WT n = 5, *P53* cKO n = 7, *BubR1* cKO n = 5, dcKO n = 5. ANOVA^CUX1^ F = 478.3, df = 3.18, P = <0.0001; Adjusted P^WT-p53 CKO^ = 0.1933, Adjusted P^WT−BUBR1 CKO^ = <0.0001, P^WT−DCKO^ = <0.0001, Adjusted P^P53 CKO−BUBR1 cKO^ = <0.0001, Adjusted P^P53 CKO−DCKO^ = <0.0001, Adjusted P^BUBR1−DCKO^ = 0.0111). Data is presented as the mean ±SEM and analyzed by one-way ANOVA followed by a posthoc Tukey test.

Because we observed a partial preservation effect in cortical cells via genetic deletion of *Trp53* during neurogenesis, we next investigated whether *Trp53* deletion could improve microcephaly. We analyzed the cortical phenotype at postnatal day (P)21 for all four genotypes. At P21, *BubR1* cKO mice are smaller with significantly shorter body lengths and reduced cortical surface area (Figure 6). Because the conditional allele was generated in a *BubR1* hypomorphic allele background (Simmons et al., 2019), the whole body possesses reduced BubR1 levels, leading to smaller body size. Of note, cortex size is only decreased when BubR1 level is further eliminated by Cre-mediated cortex specific deletion. While *Trp53* deletion alone does not alter the size or morphology of the cortex, dcKO mice display reduced body sizes and cortical surface area as in *BubR1* cKO mice (Figure 6). Through histological analyses, we observed that BubR1 loss results in a severe decrease in cortical thickness and ventricular surface length consistent with previous research (Figures 7A–C) (Simmons et al., 2019). Interestingly, when *Trp53* was concurrently deleted in the *BubR1* cKO cortex, ventricular surface length was mildly but significantly improved while cortical thickness was unchanged, as in embryonic cortex analyses (Figures 7A–C).

Because cortical thickness was not rescued in dcKO cortices, it is likely that the number of cortical layer neurons are not restored. However, it is also possible that the composition of layer specific neurons may differ in dcKO cortices compared to *BubR1* cKO brains. To assess this, we examined layer-specific neuron numbers after labeling early-born neurons with CTIP2 and FOXP2, and late-born neurons with CUX1. As expected, *Trp53* deletion alone did not alter cortical cell numbers and cortical lamination, while loss of BubR1 resulted in a significant reduction of neurons from every cortical layer (Figures 7D–G). *Trp53* co-deletion significantly rescued the number of early-born neurons labeled with FOXP2 (Figures 7D, G). However, there was no improvement in early-born neurons labeled with CTIP2, and only mild improvement in late-born neuron numbers (CUX1^+^) at P21 (Figures 7E–G). The significant but minimal rescue effects of *Trp53* co-deletion on cortical neuron numbers is consistent with the observed lack of improvement in cortical thickness in dcKO animals. Although the alternative cell death pathway may be a delayed and inefficient mechanism, our results demonstrate that the majority of cells which should be eliminated are eventually removed in the absence of P53. We propose that genomic anomalies and unrepaired DNA damage accumulation are not compatible with survival. In the absence of P53, alternative death mechanisms including P53-independent apoptosis would be responsible for the death of unfit cells.

**FIGURE 8:**
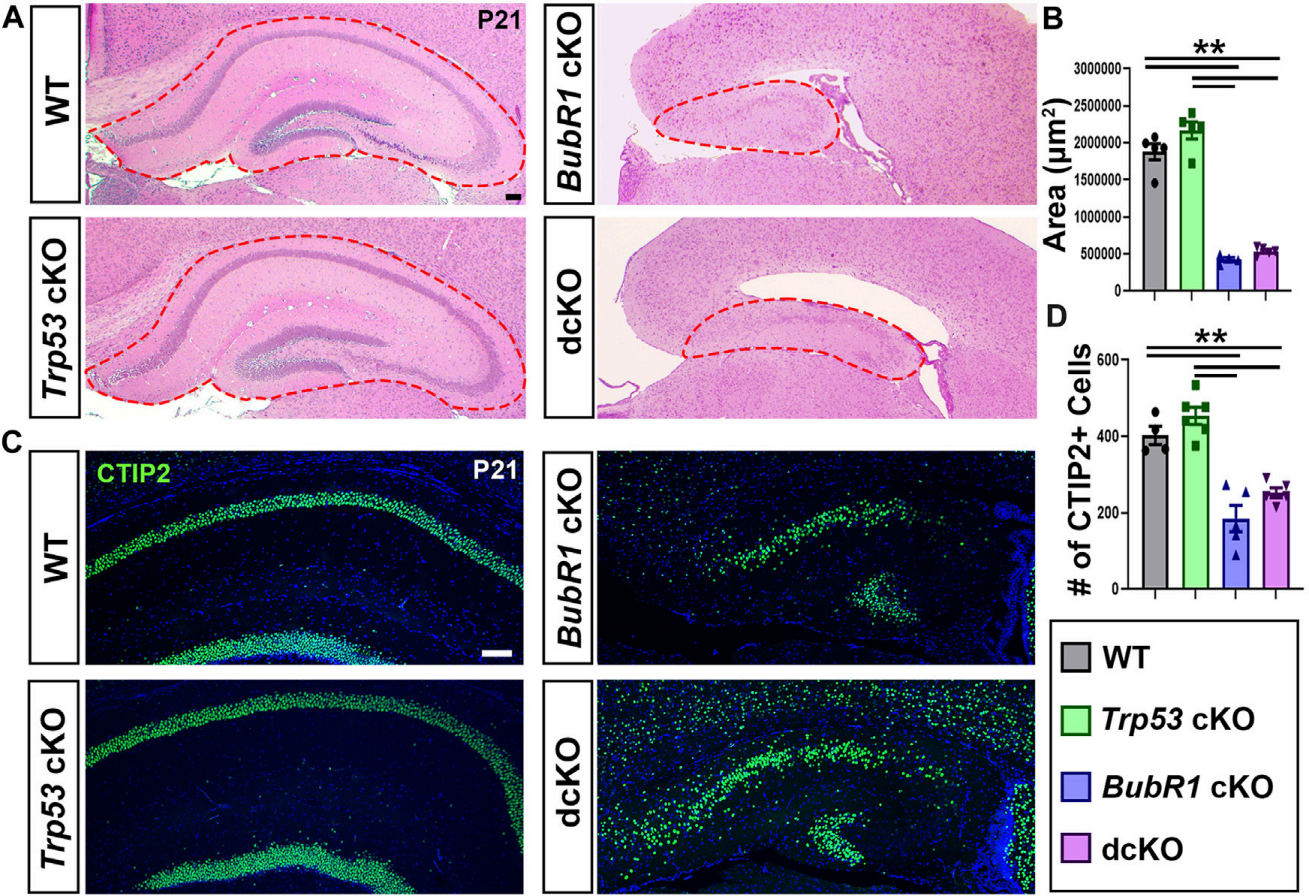
*Trp53* co-deletion does not significantly rescue hippocampal reduction in *BubR1* cKO at P21. **(A)** Hematoxylin and Eosin staining and shows that the size of the hippocampus is reduced in both *BubR1* cKO and dcKO animals. Scale bar = 100 μm. **(B)** Quantification of hippocampal area shows that hippocampal size is significantly reduced by BubR1 loss and not restored by subsequent *Trp53* co-deletion. WT n = 5, *P53* cKO n = 5, *BubR1* cKO n = 4, dcKO n = 5. (ANOVA^AREA^ F = 107.9, df = 3.15, P = <0.0001; Adjusted P^WT-p53 CKO^ = 0.1147, Adjusted P^WT−BUBR1 CKO^ =<0.0001, Adjusted P^WT−DCKO^ = <0.0001, Adjusted P^P53 CKO−BUBR1 cKO^ = <0.0001, Adjusted P^P53 CKO−DCKO^ = <0.0001, Adjusted P^BUBR1−DCKO^ = 0.8009). **(C, D)** Representative images and quantification of CTIP2^+^ neurons in the hippocampal CA1/CA2 region at P21 show a significant reduction in cell numbers caused by BubR1 loss that is unimproved in the dcKO brain. Scale bar = 100 μm. WT n = 4, *P53* cKO n = 6, *BubR1* cKO n = 5, dcKO n = 5. (ANOVA^CTIP2^ F = 26.76, df = 3.16, P = <0.0001; Adjusted P^WT-p53 CKO^ = 0.4990, Adjusted P^WT−BUBR1 CKO^ = 0.0001, Adjusted P^WT−DCKO^ = 0.0049, Adjusted P^P53 CKO−BUBR1 cKO^ = <0.0001, Adjusted P^P53 CKO−DCKO^ = 0.0001, Adjusted P^BUBR1−DCKO^ = 0.2595). Data are presented as the mean±SEM and analyzed by one-way ANOVA followed by a posthoc Tukey test.

Although we observed a mild increase in cortical neurons in the dcKO cortex compared to *BubR1* cKO, hippocampal development was not improved by *Trp53* deletion at P21. *BubR1* cKO mice display a morphologically distinct hippocampus with a significantly smaller area than in WT or *Trp53* cKO brains. The reduced hippocampal size found in *BubR1* cKO brains was not rescued by *Trp53* co-deletion (Figures 8A, B). We further quantified the number of neurons within the CA1/CA2 region of the hippocampus by labeling with CTIP2. A significant reduction in the number of CTIP2^+^ hippocampal neurons observed in BubR1 loss was also not improved by *Trp53* co-deletion (Figures 8C, D). Although we have not studied hippocampal developmental defects in the *BubR1* cKO and dcKO, it is intriguing that CTIP2^+^ granule neurons display a disorganized distribution that is especially prominent in dcKO brains. It is possible that insufficient or abnormal genomic content may contribute to defective neuronal migration. Because we observed dispersed cortical progenitors in the dcKO cortex, it is possible that ectopically located progenitors may contribute to the lamina defects found in the hippocampus. Furthermore, the failure of *BubR1* cKO brains to develop ependymal cells is not recovered in dcKO brains (data not shown). Overall, this work demonstrates only minimal improvement in the microcephaly phenotype when P53 is lost in the *BubR1* cKO cortex, suggesting that P53-mediated cortical cell loss is not the major contributor to BubR1-related microcephaly pathogenesis.

## 4 Discussion

Because many microcephaly-causing genes encode proteins that are involved in faithful mitosis, studying how mitotic defects lead to microcephaly builds a greater understanding of overall microcephaly pathogenesis (Frank et al., 2000; McConnell et al., 2004; Silver et al., 2010; Chen et al., 2014; Insolera et al., 2014; Faheem et al., 2015; Marjanovic et al., 2015; Mao et al., 2016; Bianchi et al., 2017; Sgourdou et al., 2017; Little and Dwyer, 2019; Shi et al., 2019; McNeely and Dwyer, 2020; Phan et al., 2021; White et al., 2021; Martins et al., 2022; Sterling et al., 2023). In this study, cellular analysis of *BubR1* cKO cortices identified a spectrum of structural abnormalities in chromosomes that are caused by the functional loss of BubR1. By imaging mitotic cells which are enriched at the ventricular surface in *en face* apical explants, we observed lagging chromosomes, DNA bridges, and micronuclei in *BubR1* cKO neural progenitors. Premature separation of chromatids is a hallmark of BubR1-related MVA but does not always produce chromosome structural defects (Garcia-Castillo et al., 2008; Soto et al., 2017). Furthermore, the absence of structural abnormalities in whole chromosome aneuploidy was previously shown not to induce P53 activation and cell death (Soto et al., 2017). Because dysfunctional mitotic machinery including disoriented spindle formation and kinetochore failure can produce chromosomal structure abnormalities, BubR1 function in kinetochore integrity may be critical in microcephaly pathogenesis (Ha et al., 2007; Ganem and Pellman, 2012). BubR1 has an increasingly recognized role in establishing kinetochore interaction with the mitotic spindle and recruiting PP2A to the kinetochore to antagonize mitotic phosphorylation (Burke and Stukenberg, 2008; Braga et al., 2020). Therefore, our studies demonstrating structural chromosome aberrations in the *BubR1* cKO suggest that microcephaly pathogenesis is owing to the loss of BubR1 function required for kinetochore integrity, which causes P53 activation and subsequent massive cell death.

We identified P53 activation in *BubR1* cKO cortices via elevated P21 expression and activated microglia, consistent with the idea that mitotic disruption and DNA damage activate P53. Our findings in this study demonstrate that the role of P53 activation in the *BubR1* cKO cortex is to initiate apoptotic cell death, consistent with other microcephaly animal models. Interestingly, unlike other models, we found a significant number of apoptotic cells remaining after P53 elimination, indicating the presence of a P53-independent cell death pathway mediating apoptosis in the *BubR1* mutants. For example, P73 and P63 can mediate apoptosis in the absence of P53 (Irwin and Miller, 2004; Melino, 2011; Fisher et al., 2020; Rozenberg et al., 2021; Wang et al., 2021). In particular, P73 is known to be expressed in cortical cells (Irwin and Miller, 2004; Rozenberg et al., 2021). Alternatively, JNK is a known mediator of the aneuploidy-induced P53-independent apoptotic pathway in *Drosophila* (Dhanasekaran and Reddy, 2017; Gerlach et al., 2018). We therefore postulated that apoptosis may be mediated through these potential factors in our dcKO model to eliminate cells with compromised genomic content in the absence of P53. However, as P53-independent apoptosis is shown to be less efficient (Aubrey et al., 2017), our observation of fewer CC3 and TUNEL^+^ cells in dcKO cortices compared to *BubR1* cKO brains may be due to the diminished efficiency of P53-independent cell death.

The relative inefficiency of P53-independent apoptosis poses an interesting question about whether P53 elimination has beneficial effects on microcephaly resulting from BubR1 loss. Our data showed that at the middle of active neurogenesis, P53 elimination causes a substantial rescue of ventricular length and the number of TBR2^+^ intermediate basal progenitor, and a partial rescue of the number of PAX6^+^ and SOX9^+^ aRG cells. However, we found minimal rescue effects at later stages, suggesting that most genomically unfit cells are cleared by P53-independent pathways resulting in apoptosis and necrosis. Future studies are necessary to distinguish the potential pathways that lead to cell death after BubR1 loss. This study provides new insights into the existence of P53-independent cell death in BubR1-deficient cortical cells when P53 is no longer available.

While many microcephaly models display apoptotic cell death that is mediated by P53 activation, our study demonstrates that P53 elimination minimally restores brain size in *BubR1* cKO mice. Distinguishing P53 activation as a molecular mechanism for microcephaly pathology in various models provides important insight into cellular defects linked to P53 activation. For instance, when P53 activation is directly associated with mitotic defects, genetic deletion or inhibition of P53 can substantially rescue the microcephaly phenotype (Sterling et al., 2023). Alternatively, when P53 elimination solely promotes cell survival, a variety of outcomes can be observed. Unfit cells may be removed by alternative cell death mechanisms, or defective cells survive and are observed at later stages (Yang et al., 2003; Peterson et al., 2012; Lang et al., 2016; Shi et al., 2019; Moujalled et al., 2021). In this study, the minimal rescue of cell numbers achieved by deletion of *Trp53* in *BubR1* cKO mice supports the idea that alternative cell death pathways eliminate unfit cells. In a similar case, cerebellar hypoplasia caused by loss of the DNA damage repair protein ATR is not rescued by *Trp53* deletion (Lang et al., 2016). Likewise, when microcephaly is caused by loss of the minor spliceosome protein Rnu11, *Trp53* co-deletion does not rescue microcephaly (White et al., 2021). Instead, Rnu11 deficiency causes cell cycle disruption that is exacerbated by the subsequent removal of P53. Interestingly, Knl1 deletion alone allows for animal survival, but the persistence of defective cells in *Knl1* and *Trp53* double mutants causes postnatal lethality before weaning age (Shi et al., 2019). Our results show minimal rescue effects but no further compromised viability by *Trp53* deletion, highlighting the importance of P53-independent cell death pathways for clearing aberrant cells.

In this study, BubR1 cortical deficiency results in many cortical cells and mitotic cells with γH2AX^+^ DNA damage accumulation that is not prevented by P53 removal. Interestingly, in mitotic cells, DNA damage was detected in prophase but not beyond. Our previous study found a reduced fraction of metaphase cells and an increased fraction of prophase cells among total mitotic cells in *BubR1* cKO brains, suggesting that metaphase was shortened by the compromised mitotic checkpoint or prophase was lengthened to allow for the DNA damage response (Simmons et al., 2019). The data in this study suggests the possibility that prophase cells with DNA damage may not proceed through mitosis and could undergo mitotic catastrophe resulting in elimination of these unfit cells. In future studies, the use of time-lapse imaging will be important to distinguish between the possibility of lengthened prophase or unchecked metaphase in BubR1-deficient cells. It will also be important to address the question of how BubR1 loss generates extensive DNA damage. Chromosome segregation errors can result in genomic instability in a variety of ways (Yang et al., 2003; Ganem and Pellman, 2012; Peterson et al., 2012; Luzhna et al., 2013; Ohashi et al., 2015; Rohrback et al., 2018). For example, lagging chromosomes can be trapped and damaged by cleavage furrow progression or translocated to other chromosomes (Ganem and Pellman, 2012). BubR1 is also known to play a role in the DNA damage response (Ha et al., 2007; Baker et al., 2012; Weaver et al., 2016). Elevated BubR1 expression in cancer cells with DNA damage induced by chemoradiation therapy increased non homologous end joining repair (NHEJ) and promoted cell survival (Komura et al., 2021). However, this study cannot distinguish whether BubR1 function is directly required to prevent DNA damage by promoting faithful mitosis to prevent aneuploidy, or if BubR1 is active in repair mechanisms. Importantly, our results show the presence of DNA damage that may have a causative role in P53 activation and cell death.

Overall, our study shows that chromosome structural defects result from BubR1 deficiency in cortical progenitors, providing a potential explanation for massive cortical cell death. Our results also demonstrate that P53 activation occurs in the developing cortex when BubR1 is removed, but loss of cortical volume and cell numbers are only mildly improved by *Trp53* deletion in *BubR1* cKO brains. This inability to rescue BubR1-dependent cortical cell loss by *Trp53* deletion argues the robustness of P53-independent cell death pathways. We therefore propose a model in which genomically unfit cells are generated through aneuploidy, chromosome structural defects and DNA damage after BubR1 loss. Ultimately, these unfit cells can eventually be cleared even in the absence of P53-dependent pathways.

## Data Availability

The original contributions presented in the study are included in the article/Supplementary Material, further inquiries can be directed to the corresponding author.
